# History of depression and risk of hyperemesis gravidarum: a population-based cohort study

**DOI:** 10.1007/s00737-016-0713-6

**Published:** 2017-01-07

**Authors:** Helena Kames Kjeldgaard, Malin Eberhard-Gran, Jūratė Šaltytė Benth, Hedvig Nordeng, Åse Vigdis Vikanes

**Affiliations:** 1 Health Services Research Unit, Akershus University Hospital, Post Box 1000, 1478 Lørenskog, Norway; 2 Institute of Clinical Medicine, Campus Ahus, University of Oslo, Lørenskog, Norway; 3 Domain for Mental and Physical Health, Norwegian Institute of Public Health, Oslo, Norway; 4 PharmacoEpidemiology & Drug Safety Research Group, Department of Pharmacy, School of Pharmacy, University of Oslo, Oslo, Norway; 5 The Intervention Centre, Oslo University Hospital, Oslo, Norway

**Keywords:** Depression, Hyperemesis gravidarum, Mental health, Nausea and vomiting, Norwegian Mother and Child Cohort Study

## Abstract

Hyperemesis gravidarum (HG) is a pregnancy condition characterised by debilitating nausea and vomiting. HG has been associated with depression during pregnancy but the direction of the association remains unclear. The aim of this study was to assess whether previous depression is associated with HG. This is a population-based pregnancy cohort study using data from The Norwegian Mother and Child Cohort Study. The study reviewed 731 pregnancies with HG and 81,055 pregnancies without. Logistic regression analyses were performed to examine the association between a lifetime history of depression and hyperemesis gravidarum. Odds ratios were adjusted for symptoms of current depression, maternal age, parity, body mass index, smoking, sex of the child, education and pelvic girdle pain. A lifetime history of depression was associated with higher odds for hyperemesis gravidarum (aOR = 1.49, 95% CI (1.23; 1.79)). Two thirds of women with hyperemesis gravidarum had neither a history of depression nor symptoms of current depression, and 1.2% of women with a history of depression developed HG. A lifetime history of depression increased the risk of HG. However, given the fact that only 1.2% of women with a history of depression developed HG and that the majority of women with HG had no symptoms of depression, depression does not seem to be a main driver in the aetiology of HG.

## Introduction

Nausea and vomiting in pregnancy (NVP) is common and affects up to 80% of all pregnancies (Gadsby et al. 1993). Unlike NVP, hyperemesis gravidarum (HG) is characterised by severe, debilitating symptoms. The International Classification of Diseases (ICD-10) describes HG as excessive vomiting starting before the 22nd week of gestation with (severe HG) or without (mild HG) metabolic disturbances (World Health Organization 2004). Although estimated to affect 0.3 to 2% of all pregnancies (Eliakim et al. 2000), HG is a primary reason for sick leave (Dorheim et al. 2013) and hospitalisation during pregnancy (Gazmararian et al. 2002). The aetiology and the pathogenesis of HG are unclear, and it remains unknown whether NVP and HG are independent conditions or if HG represents the extreme of a continuum of NVP.

HG has historically been explained by a variety of psychological mechanisms that have been subjected to stigma (Fairweather 1968). Other hypotheses have been proposed, including genetic components (Corey et al. 1992; Fejzo et al. 2008), endocrine factors and *Helicobacter pylori* infection, but none of these have proven sufficient to explain HG (Verberg et al. 2005). Although, HG is today considered a disease of unclear pathophysiology (Grooten et al. 2015), clinical practice still includes evaluation of hyperemetic women for psychiatric disease (Kim et al. 2009). Women with HG report lack of support from their healthcare providers (Heitmann et al. 2016; Poursharif et al. 2008), which may have severe consequences such as termination of pregnancy and psychological sequelae (Poursharif et al. 2008; Poursharif et al. 2007).

HG has consistently been associated with mental distress such as depression and anxiety. Previous studies are, however, often small with a medium to high risk of bias (Mitchell-Jones et al. 2016) or have limited availability of co-variates (Fell et al. 2006; Seng et al. 2007). Prior research has mainly focused on the association between anxiety/depression and HG during pregnancy, whereas the effect of anxiety/depression prior to pregnancy remains to be elucidated. Furthermore, few studies have used reliable psychometric instruments to assess anxiety/depression before pregnancy, rendering causal inferences difficult (Fell et al. 2006; Seng et al. 2007). Thus, a key question remains of whether mental distress leads to HG or HG leads to mental distress.

The aim of the present study was to assess whether a lifetime history of depression is associated with HG. The Norwegian Mother and Child Cohort Study, comprising more than 100,000 pregnancies, provides a unique opportunity to explore this association.

## Materials and methods

### Study design and study population

From 1998 to 2008, all pregnant women scheduled to give birth at 50 of Norway’s 52 hospitals with maternity units received a postal invitation to participate in The Norwegian Mother and Child Cohort Study (MoBa) together with appointments for routine ultrasound examination at around week 17 of pregnancy. All participants signed an informed consent form (Magnus et al. 2016; Magnus et al. 2006). MoBa was approved by the Regional Committee for Medical Research Ethics and by the Norwegian Data Protection Authority. The protocol for the current study was submitted to the Norwegian Institute of Public Health, who, upon approval, supplied the researchers of this study with anonymised data through contract (PDB 1527, www.fhi.no/moba).

The current study is based on version 8 of the quality-assured data files linked to the Medical Birth Registry of Norway (MBRN). The MBRN is based on the compulsory notification of every birth or late abortion in Norway from the 16th week of gestation, including information regarding pregnancy-related complications (Irgens 2000). Approximately 40% of the invited women participated, and each pregnancy was registered with a unique identification number (Magnus et al. 2006).

The analyses of the current study are based on two questionnaires distributed in pregnancy week 17 (Q1) and week 30 (Q2). Q1 covers background factors including previous pregnancies, medical history before and during pregnancy, medication; occupation, lifestyle habits and mental health. Q2 provides information about the mental and physical health at this stage of pregnancy as well as changes in work situation and habits. English translations of the questionnaires can be found at http://www.fhi.no/moba.

We included all singleton pregnancies (*n* = 112,288). We excluded women with missing information on history of depression (*n* = 3605), symptoms of depression at the 17th gestational week, hospitalisation (*n* = 19,275), sex of the child (*n* = 207) and education (*n* = 15,707). Some women had missing values on more than one variable. The final sample comprised 81.786, 72.8% of the total sample.

### Variables

In accordance with previous studies on MoBa data (Vikanes et al. 2010, 2013), HG was defined as prolonged nausea and vomiting leading to hospitalisation before the 25th gestational week as reported in Q2 (week 30). This definition was chosen in order to clearly separate HG from normal levels of NVP.

The main predictor was a lifetime history of depression, measured by the Kendler’s lifetime major depression scale (KLTDS). The KLTDS was defined using five of the nine symptomatic criteria for major depression in DSM-III-R: Have you ever experienced the following for a continuous period of 2 weeks or more: (1) felt depressed, sad; (2) had problems with appetite or eaten too much; (3) been bothered by feeling weaker or a lack of energy; (4) really blamed yourself and felt worthless and (5) had problems with concentration or had problems making decisions. The response to each question was yes or no. A history of depression was defined as present if a minimum of three of the five symptoms and sad mood were reported to occur simultaneously for more than 2 weeks (Kendler et al. 1993).

A five-item short version (SCL-5) of the Hopkins Symptom Checklist-25 (SCL-25) was used as a proxy for current depression in pregnancy week 17. The SCL-5 is highly correlated with the SCL-25 (correlation coefficient of 0.92) (Tambs and Moum 1993) and consists of the following questions: Have you been bothered by any of the following during the last 2 weeks: (1) feeling fearful, (2) nervousness or shakiness inside, (3) feeling hopeless about the future, (4) feeling blue and (5) worrying too much about things. The response categories ranged from ‘not bothered’ to ‘very bothered’ (range 1–4), with a maximum total score of 20. Symptoms of current depression were defined as a mean score >2 (Strand et al. 2003), which has been shown to provide the same prevalence estimate of a depressive disorder as the Composite International Diagnostic Interview (Robins et al. 1988; Sandanger et al. 1998). Missing values in the dichotomised version of the SCL-5 were handled as follows. First, the average score on existing items was calculated for each case if at least three of five questions were answered. If the average of the existing items was clearly above or below the cut-off and could not be affected by imputation of missing values, it was dichotomised to zero or one, as appropriate. Imputation was not performed in cases where the average score was not uniquely defining the value above or below cut-off. Altogether, *N* = 18 cases were imputed.

Co-variates and possible confounders obtained from the MBRN included sex of the child (Rashid et al. 2012), maternal age and parity. Co-variates and possible confounders obtained from MoBa Q1 were socio-economic status, BMI and smoking (Vikanes et al. 2010). Pelvic girdle pain was obtained from MoBa Q2 (Bjelland et al. 2013; Chortatos et al. 2015). Regarding parity, women were dichotomised as either primiparous or multiparous. Education was used as a proxy for socio-economic status, and length of education (in years) was divided into three categories. Pre-pregnancy body mass index (BMI) was calculated as weight/height^2^. Women shorter than 120 cm (*n* = 199) and women weighing more than 150 kg or less than 40 kg were excluded (*n* = 58). Also, those reporting reduction in weight by more than 20 kg or increase in weight by more than 50 kg since the start of pregnancy were excluded (*n* = 65). Smoking was assessed as a yes/no response to the question ‘did you smoke 3 months before pregnancy’ (Vikanes et al. 2010). Pelvic girdle pain was defined as pain in the anterior pelvis and on both sides in the posterior pelvis (Bjelland et al. 2013).

Other co-variates including *H. pylori* infection (Li et al. 2015), gastrointestinal disorders, rheumatoid arthritis, pre-eclampsia, chronic hypertension, type 1 diabetes, asthma (Bolin et al. 2013; Fell et al. 2006; Jorgensen et al. 2012), eating disorders (Torgersen et al. 2008) and ethnicity (Vikanes et al. 2008) were considered but not included in the final analysis due to a small number of women with these disorders in the HG group. Thyroid disease was not included in the analysis as the questionnaire form does not allow differentiation between hypothyroid and hyperthyroid disease.

### Statistical analysis

Demographic and clinical characteristics among women with and without HG and for the entire sample were presented as frequencies and percentages or means and standard deviations (SD).

To assess the association between a lifetime history of depression and HG, a logistic regression model was estimated. Due to multiple births, some women had several recordings in the data set. According to the intra-women correlation coefficient, there was some degree of clustering detected. Thus, the generalised estimating equations (GEE) model correctly adjusting the estimates for intra-women correlations was fitted.

A number of potential predictors and confounders were considered. In order to test our hypotheses, a data splitting approach was applied (Dahl et al. 2008). According to this approach, the data set was split into two random parts containing approximately 30% (part I) and 70% (part II) of observations. Splitting was performed within stratas defined by several key variables. Part I (pilot) was used to construct a model for HG. Only predictors significant at the 5% level or those otherwise considered important were left in the model estimated on pilot data. The hypothesis testing was then performed on part II (test) data. Only the results with *P* values below 0.05 in the test data analyses were accepted as significant, regardless of significance level in the pilot part. Once the hypotheses were tested, the model was estimated on the entire data set to achieve most accurate estimates for the model parameters. Due to the numerous predictors considered, the level of significance was set to 0.005 when interpreting the results in the entire data set.

The interaction between BMI and smoking status was assessed and kept in the model if significant.

All analyses were performed by SPSS v 22.

## Results

Characteristics for the HG group and comparison group are presented in Table 1. The mean age of pregnant women was 30.3 years (15–47 years; SD 4.5 years) and 45% were primipara. A total of 731 (0.9%) women reported hospital admission due to HG. More than 20% (17,351/81,786) of the women reported a lifetime history of depression, whereas 6.1% (4981/81,786) reported symptoms of current depression at the 17th gestational week.

**Table 1:** Characteristics of the sample according to HG status among 81,786 women

	HG *n* (%)	No HG *n* (%)	Total *n* (%)
History of depression			
No	520 (71.1)	63,915 (78.9)	64,435 (78.8)
Yes	211 (28.9)	17,140 (21.1)	17,351 (21.2)
Symptoms of current depression			
Low score	650 (88.9)	76,155 (94.0)	76,805 (93.9)
High score	81 (11.1)	4900 (6.0)	4981 (6.1)
Parity			
Primipara	287 (39.3)	36,480 (45.0)	36,767 (45.0)
Multipara	444 (60.7)	44,575 (55.0)	45,019 (55.0)
Length of education (years)			
<12	79 (10.8)	5599 (6.9)	5678 (6.9)
13–16	536 (73.3)	56,034 (69.1)	56,570 (69.2)
>16	116 (15.9)	19,422 (24.0)	19,538 (23.9)
Smoking			
No	495 (79.5)	49,153 (69.3)	49,648 (69.4)
Yes	128 (20.5)	21,811 (30.7)	21,939 (30.6)
Sex of the child			
Boy	307 (42.0)	41,571 (51.3)	41,878 (51.2)
Girl	424 (58.0)	39,484 (48.7)	39,908 (48.8)
Pelvic girdle pain			
No	583 (79.8)	69,145 (85.3)	69,728 (85.3)
Yes	148 (20.2)	11,910(14.7)	12,058 (14.7)
	HG mean (SD)	No HG mean (SD)	Total mean (SD)
Maternal age	29.3 (4.9)	30.3 (4.5)	30.3 (4.5)
Pre-pregnancy BMI	24.5 (4.2)	24.1 (4.3)	24.1 (4.3)

In the binary logistic regression model, a lifetime history of depression was associated with higher odds for HG (unadjusted OR = 1.53, 95% CI (1.29; 1.83)). Adjusting for potential confounders including symptoms of depression in gestational week 17 did not influence our results (adjusted OR = 1.49, 95% CI (1.23; 1.79)).

Symptoms of depression at the 17th gestational week was independently associated with HG in the multivariate model (OR = 1.71, 95% CI (1.31; 2.23)). As shown on Table 2, other factors positively associated with HG included short education, female sex of the child, multiparity, younger age of the mother and pelvic girdle pain. Pre-pregnancy BMI did not differ between women with and without HG, and smoking was negatively associated with HG.

**Table 2:** Unadjusted and adjusted odds ratios (OR) with 95% confidence intervals (CI) for hyperemesis gravidarum (*n* = 611, 0.9%) among 69,864 pregnancies

	Unadjusted OR (95% CI)	*P* value	Adjusted OR (95% CI)	*P* value
History of depression				
No	1	–	1	–
Yes	1.53 (1.29; 1.83)	< 0.001	1.49 (1.23; 1.79)	<0.001
Symptoms of current depression				
Low score	1	–	1	–
High score	2.11 (1.65; 2.69)	< 0.001	1.71 (1.31; 2.23)	<0.001
Maternal age	0.94 (0.93; 0.96)	< 0.001	0.93 (0.91; 0.95)	<0.001
Parity				
Primipara	1	–	1	–
Multipara	1.24 (1.05; 1.45)	0.010	1.43 (1.20; 1.69)	<0.001
Length of education (years)				
<12	2.42 (1.76; 3.32)	< 0.001	1.91 (1.36; 2.69)	<0.001
13–16	1.64 (1.31; 2.06)	< 0.001	1.44 (1.13; 1.82)	0.003
>16	1	–	1	–
Pre-pregnancy BMI	1.03 (1.01; 1.04)	0.002	1.02 (1.00; 1.04)	0.030
Smoking				
No	1	–	1	–
Yes	0.60 (0.49; 0.72)	<0.001	0.46 (0.37; 0.56)	<0.001
Sex of the child				
Boy	1	–	1	–
Girl	1.49 (1.27; 1.75)	<0.001	1.50 (1.28; 1.76)	<0.001
Pelvic girdle pain				
No	1	–	1	–
Yes	1.52 (1.25; 1.85)	<0.001	1.30 (1.06; 1.59)	0.011

We also assessed whether women with a history of depression were more likely to be hospitalised during pregnancy in general. Among women with previous depression, 7.7% were hospitalised during pregnancy compared to 5.2% without; history of depression was associated with higher odds for hospitalisation (OR = 1.52, 95% CI (1.42; 1.62)).

**Fig. 1:**
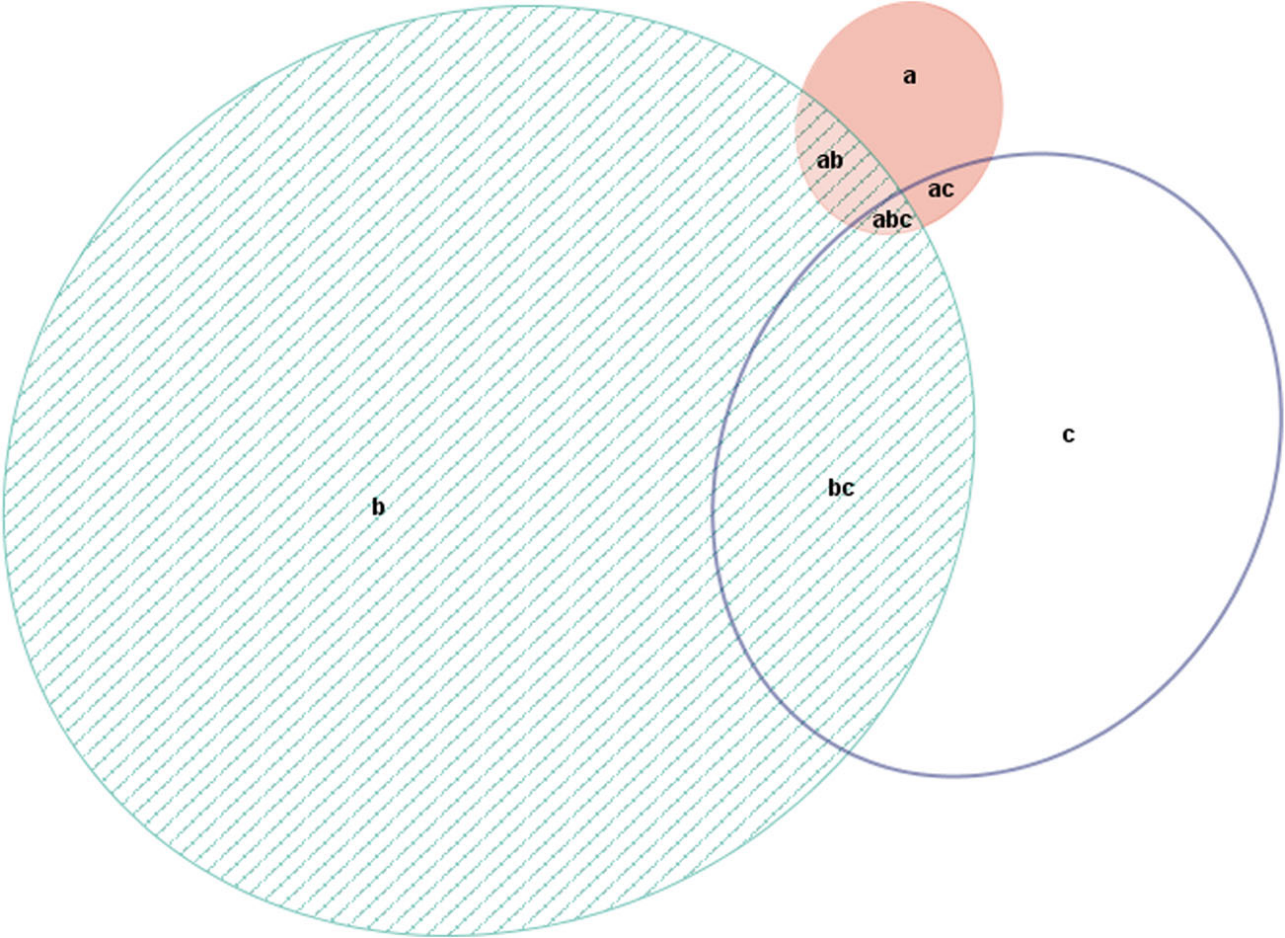
The total number of women with *a* HG (*n* = 731), *b* a history of depression (n = 17,351) and *c* symptoms of current depression (n = 4981) among 81,786 women in the Norwegian Mother and Child Cohort Study. The number of women with HG and a history of depression (*ab*, *n* = 211); with HG and symptoms of current depression (*ac*, *n* = 81) and with HG, a history of depression and symptoms of current depression (*abc*, *n* = 50). The number of women with no HG and a history of depression and symptoms of current depression (*bc*, *n* = 2910)

Although HG was positively associated with depression, the majority of women with HG (66%, 489/740) neither had a lifetime history of depression nor symptoms of depression in the 17th gestational week as shown in Fig. 1. Furthermore, only 1.2% of women with previous depression developed HG.

## Discussion

The main finding of the present study was that having a lifetime history of depression was associated with 50% higher odds for HG. The majority of women with HG did not, however, have a history of depression, and less than 2% of women with previous depression developed HG.

The results are in line with previous research. Using health insurance data from the Midwestern USA between 2000 and 2004, Seng et al. (2007) found that a diagnosis of depression before pregnancy was positively associated with HG in a population of 11,016 women, including 208 HG pregnancies (OR = 3.2, 95% CI (2.0; 5.2)). Additionally, they found that the burden of illness increased the likelihood of HG. Having had a psychiatric or somatic condition before pregnancy increased the odds for HG twofold, while having had both a psychiatric and somatic condition increased the odds fourfold. The study design permitted the identification of psychiatric diagnoses occurring before pregnancy, but information about other co-variates was limited.

Another large cohort study comprising 157,922 women, of whom 1301 had HG, was extracted from a population-based healthcare database covering all deliveries to residents of Nova Scotia, Canada, between 1988 and 2002 (Fell et al. 2006). The study revealed a fourfold higher risk of HG in women with psychiatric disease (RR = 4.1, 95% CI (3.0; 5.7)). The timing or types of psychiatric disease were not specified, but a crude RR of 2.5 with 95% CI (1.5; 4.2) was reported for HG in women with depression compared to women without depression.

On the other hand, Magtira et al. (2014) found no statistically significant differences in the prevalence of psychiatric conditions prior to the first pregnancy when comparing 84 women with recurrence of HG with 34 women with no recurrence. The authors predicted that if psychiatric symptoms positively correlate with HG, then psychiatric symptoms would correlate positively with recurrence risk. As the study was based on data from an online survey, the participating women were not randomly selected among women with HG and may therefore have had a different risk profile from the women who did not participate (Bornehag et al. 2012). Additionally, only women who had had at least two pregnancies lasting beyond the second trimester were included, which may introduce recall bias, e.g. whether psychiatric symptoms preceded pregnancy, or selection bias as women with poor psychosocial health may have been less likely to continue participation, as were women terminating their pregnancies due to HG (McDonald et al. 2013).

Given the nature of the MoBa data, we were able to explore whether symptoms of depression in the current pregnancy were independently associated with HG. Consistent with a recent meta-analysis (Mitchell-Jones et al. 2016), we found an association between HG and depression during pregnancy. Two prospective studies, both excluding women with a history of psychiatric disease, also reported that women with HG were more likely to suffer from symptoms of anxiety and depression during pregnancy compared to asymptomatic pregnant women (Aksoy et al. 2015; Pirimoglu et al. 2010). It was therefore argued that psychological distress was a consequence of HG rather than the cause (Aksoy et al. 2015).

Since hospitalisation due to prolonged NVP was a requirement for having HG in the current study, our results may have been biased by a greater likelihood of being hospitalised among women with a history of depression (Atanackovic et al. 2001). A relationship between depression and severity of NVP has previously been suggested (Kelly et al. 2001; Mazzotta et al. 2000) although other studies do not support this finding (Swallow et al. 2004; Tan et al. 2010). We therefore assessed whether women with previous depression were more likely to be hospitalised in general during pregnancy. Previous depression was associated with hospitalisation (OR = 1.52, 95% CI (1.42; 1.62)), which may have contributed to overestimating the effect of previous depression on the risk of HG. However, in Norway, only women with severe symptoms of HG, including metabolic disturbances, are hospitalised. Additionally, there is no tradition for outpatient treatment for these patients. This indicates that our sample is restricted to severe HG cases corresponding to ICD 10 code O21.1, and it is therefore unlikely that the women have been hospitalised due to depression. Given that hospital care in Norway is free of charge, it is furthermore unlikely that more socially disadvantaged women are less likely to be hospitalised.

Several studies show that a variety of somatic diseases such as pelvic girdle pain, *H. pylori* infection, thyroid disease, gastrointestinal disorders, rheumatoid arthritis, pre-eclampsia, chronic hypertension, type 1 diabetes, asthma and eating disorders are associated with higher risk of HG (Bolin et al. 2013; Fell et al. 2006; Jorgensen et al. 2012; Li et al. 2015; Seng et al. 2007; Torgersen et al. 2008). However, in the present study, the number of HG cases with these conditions was too small to explore possible influences of these conditions. Our results should be interpreted with these limitations in mind.

HG is a diagnosis by exclusion and an international consensus on the definition of HG is yet to be established, limiting comparison of previous research (Mitchell-Jones et al. 2016). The lack of consensus is a challenge for clinicians who may need to distinguish milder forms of HG from more common nausea and vomiting in pregnancy (Grooten et al. 2015). Inadequate care of women with HG may have severe consequences including therapeutic abortions, Wernicke’s encephalopathy and even death (Eliakim et al. 2000; Poursharif et al. 2007). Adverse pregnancy outcomes such as low birth weight and preterm delivery may in particular affect HG women with poor pregnancy weight gain (<7 kg) (Dodds et al. 2006). Adequate care of women with HG is thus of the utmost importance.

To our knowledge, this is the first time a large, high-quality data set enables the study of the associations between a history of depression and HG and symptoms of depression during pregnancy and HG. Our results advocate that routine psychiatric consultations of HG women may be unnecessary. Treatment should focus on relief of somatic complaints and ensure the health of the mother and child.

The large number of HG pregnancies is a major strength of the current study. Furthermore, the study covered all regions of Norway, and the prospective nature of data collection minimises the risk of recall bias. To date, more than 400 articles have been published based on MoBa data. Around 40% of the invited women participated in the study, introducing a possibility of self-selection bias. However, a recent study looking into potential bias by skewed selection of participants in MoBa found that the participant selection influenced the prevalence estimates but not the exposure outcome associations (Nilsen et al. 2009). Women known to be underrepresented in MoBa include single women, those with shorter education, those under 25 years of age, immigrants and smokers (Nilsen et al. 2009; Vikanes et al. 2010). Hospitalisation for HG was assessed retrospectively; however, recall bias is highly unlikely due to the relatively short interval between hospitalisation and reporting of HG in week 32 of pregnancy (Vikanes et al. 2010). The comparison group comprised all other pregnant women in the study, including those with complications other than HG, reducing the risk of overestimating the association between previous depression and HG.

The KLTDS and SCL-5 are the only available measures of mental health in the MoBa study. Unlike clinical interviews, the KLTDS and SCL-5 cannot be used to diagnose depression. The scales have, however, been developed and validated to measure symptoms of depression in population studies. Extensive questionnaire studies with a broad scope such as the MoBa study often have a shortage of space for the original lengthy psychometric instruments, and short versions may be useful to improve response rates. While the short versions affect the measurement precision, the precision remains sufficient for epidemiological purposes (Strand et al. 2003; Tambs and Moum 1993; Tambs and Røysamb 2014).

The fact that a history of depression was not measured before pregnancy is a limitation of our study. Women responded to the KLTDS in gestational week 17, which for most women with HG is after the onset of severe nausea and vomiting. This may have affected their response. In our analyses, we therefore adjusted for symptoms of current depression at the 17th gestational week to quantify the direct effect of a previous depression on HG. The effect estimates changed only slightly in the adjusted model indicating that KLTDS and SCL-5 cover different aspects of women’s mental health in relation to HG.

## Conclusion

In conclusion, a lifetime history of depression increased the odds for hospitalisation for HG by approximately 50%. However, two thirds of women with HG had neither a history of depression nor symptoms of depression at the 17th gestational week. Given the fact that only 1.2% of women with previous depression developed HG, depression does not appear to be a main driver in the aetiology and pathogenesis of HG. Our results advocate that routine psychiatric consultations may be unnecessary.
